# A Long Short-Term Memory neural network for the detection of epileptiform spikes and high frequency oscillations

**DOI:** 10.1038/s41598-019-55861-w

**Published:** 2019-12-18

**Authors:** A. V. Medvedev, G. I. Agoureeva, A. M. Murro

**Affiliations:** 1 Center for Functional and Molecular Imaging & Department of Neurology, Georgetown University, Washington, DC USA; 2 Faculty of Science and Engineering, Flinders University of South Australia (Retiree), Adelaide, SA Australia; 3 Department of Neurology, Medical College of Georgia, Augusta, GA USA

**Keywords:** Learning algorithms, Network models, Diagnostic markers, Epilepsy

## Abstract

Over the last two decades, the evidence has been growing that in addition to epileptic spikes high frequency oscillations (HFOs) are important biomarkers of epileptogenic tissue. New methods of artificial intelligence such as deep learning neural networks can provide additional tools for automated analysis of EEG. Here we present a Long Short-Term Memory neural network for detection of spikes, ripples and ripples-on-spikes (RonS). We used intracranial EEG (iEEG) from two independent datasets. First dataset (7 patients) was used for network training and testing. The second dataset (5 patients) was used for cross-institutional validation. 1000 events of each class (spike, RonS, ripple and baseline) were selected from the candidates initially found using a novel threshold method. Network training was performed using random selections of 50–500 events (per class) from all patients from the 1^st^ dataset. This ‘global’ network was then tested on other events for each patient from both datasets. The network was able to detect events with a good generalisability namely, with total accuracy and specificity for each class exceeding 90% in all cases, and sensitivity less than 86% in only two cases (82.5% for spikes in one patient and 81.9% for ripples in another patient). The deep learning networks can significantly accelerate the analysis of iEEG data and increase their diagnostic value which may improve surgical outcome in patients with localization-related intractable epilepsy.

## Introduction

Pathophysiological mechanisms of epilepsy are poorly understood and despite any available treatment approximately 30% of patients still have recurrent seizures. Epileptogenesis occurs at different levels of neuronal organization from cellular and synaptic mechanisms to the level of neural networks and brain systems. The immediate cause of an epileptic seizure is an extensive electrical activity of neurons which manifests itself in marked changes in the on-going activity of the brain. These changes are seen in electroencephalogram (EEG) as specific patterns of activity which include high amplitude sharp (fast) and slow waveforms. Sharp events with duration from 20–70 milliseconds are called spikes and they can be also recorded in background (i.e., quiet) EEG between seizures. Those interictal spikes have been historically considered as a hallmark of epilepsy manifesting pathological tissue prone to the development of epileptiform discharges as well as electrographic and behavioural seizures. Over the last two decades, however, the evidence has been growing that, in addition to traditional epileptiform events such as spikes and spike-wave discharges, high frequency oscillations (HFOs) may be important electrophysiological markers of the epileptic process. In general, high frequency (HF) activity of the brain occupies frequencies above the historical Berger bands that is, above 30 Hz, and are referred to as gamma activity (30–120 Hz), ripples (120–250 Hz) and fast ripples (250–500 Hz). Importantly, all these types of HF activity are observed in both physiological and pathological conditions with a varying presence across those conditions. It is now well-established that gamma oscillations underlie many perceptive, cognitive and motor functions with good anatomical, functional and temporal specificity^1^ (for review, see^2^). Thus, gamma activity is commonly considered as physiological^3^. Fast ripples have been suggested to preferentially occur in epileptogenic tissue^4^ and therefore are mainly studied as pathological oscillations. Ripples, in turn, can be observed during slow wave sleep in various brain regions as well as in the epileptogenic zone, and therefore can be considered either physiological or pathological depending on the functional state and anatomical location^3^. Several research groups have argued that detection and quantitative analysis of ripples and fast ripples is necessary for a more accurate localization of epileptogenic tissue which may improve surgical outcome in patients with localization-related intractable epilepsy^5–13^. Despite their promise as a new biomarker of epileptogenic tissue, the implementation of quantitative evaluation of HFOs into clinical practice remains a challenging task due to the technical difficulties to detect those types of electrical activity. HFOs have a relatively small amplitude and a low signal-to-noise ratio and also require high sampling rates. Even with the use of intracranial EEG (iEEG) recordings which have a higher fidelity and less myogenic artefacts compared to the scalp EEG and magnetoencephalography (MEG), the task of finding HFOs during visual inspection of several-hour and up to 100-channel iEEG record is extremely burdensome. It has been estimated that it takes about 10 h to visually mark HFOs in a 10-channel 10-min recording^11^. Moreover, the individual marking of HFOs even by experts remains to be subjective due to the lack of widely accepted guidelines on the HFO spectral and temporal features, and this leads to a relatively low agreement in the inter-rater estimates^14^.

Because of the challenges described above, there is a strong need to develop analytical techniques which would allow automatic detection of HFOs in electromagnetic activity of the brain (EEG/MEG/iEEG). The development of automated algorithms has started about two decades ago^7^ and up to date a good number of such algorithms have been published (for comprehensive reviews, see^11^ and^3^). The majority of those algorithms are based on the following steps. The first step is to apply bandpass or highpass (HP) filtering of the signal within the frequency range of interest e.g., 80–250 Hz for ripples and >250 Hz for fast ripples. The second step is to find events which are noticeably different from (i.e., larger than) the baseline activity. This is achieved by an introduction of a threshold used to detect events which are above the threshold and thus ‘stand out’ from baseline activity. The threshold is usually based on spectral power in the frequency domain or amplitude squared in the time domain (Root Mean Square, RMS) or some other related characteristics such as Line Length. Four algorithms^7,10,11,15^ have been more commonly used by different research groups and recently were implemented within two software toolboxes designed specifically for the HFO analysis^3,16^. These algorithms are based on supervised detection methods and have the reported sensitivity higher than 80%. Despite the existence of so many automated algorithms to detect HFOs, they are still suboptimal due to several reasons. First, the choice of threshold remains somewhat subjective and different groups use different threshold values (e.g., 2–5 standard deviations from the mean power/RMS of the signal). The definition of threshold also requires a proper selection of baseline activity because standard deviation calculated over the whole record may be inflated by the presence of relatively rare but higher-than-baseline events such as HFOs. This would lead to masking of HFO events and the larger number of misses (false negative errors). To overcome this problem, the MNI method, for example, uses the analytic procedure to detect baseline activity first^11^.

Over the last few years, new methods of artificial intelligence such as deep learning (DL) are being developed with various applications in research and clinical practice^17,18^. Deep learning refers to artificial neural networks which in turn belong to a broader class of machine learning algorithms. They represent a ‘general purpose computer’ in a sense that they can process any type of data through supervised or unsupervised learning. An important property of the DL networks is that they can extract and learn high-level abstracted features from various data and thus do not necessarily need features selected by a ‘human trainer’^19^.

In this study we used a Long Short-Term Memory (LSTM) neural network for the detection of epileptiform events. The LSTM network was first developed by Hochreiter and Schmidhuber^20^ as a special type of the deep neural network architecture being able to correlate stimuli/events separated by time and to learn long-term dependencies in the input signal. Because of this ‘short-term’ memory of these networks, they are well suited to analyse time series data. The LSTM network is an extended class of Recurrent Neural Networks (RNNs) where the main components of a hidden layer are ‘cells’. The cell behaviour is controlled by three gates (input gate, forget gate and output gate). These gates determine the cell learning rate, can solve the problem of vanishing gradients and provide a better control of what information is kept or forgotten across longer times.

Here we apply the LSTM network for the detection of three specific electrographic events namely, individual epileptic spikes, ripples and ‘ripples-on-spike’ (RonS). We used the intracranial datasets from two different sources for a ‘between-institutions’ validation and demonstrate that the LSTM network can achieve high values of accuracy, sensitivity and specificity for the detection of spikes and HFOs as well as an excellent ‘between-institutions’ generalisability when trained on diverse data from seven patients.

## Methods

### Patient information and intracranial recordings

We used intracranial EEG records from 12 patients with drug-resistant epilepsy obtained strictly for clinical purposes during their evaluation for surgery. Records from 7 patients were taken from the open access database ieeg.org (files from the Mayo Clinic; the MC data set). The second dataset (5 patients) was obtained from the Medical College of Georgia, Augusta University Health (the AUH dataset). The study was carried out in accordance with the relevant ethical guidelines and regulations and was approved by the Georgetown-MedStar Institutional Review Board and the Medical College of Georgia’s Institutional Review Board. Informed consent was obtained for a secondary analysis of patients’ de-identified iEEG records. The MC dataset was used for all network training, cross-validation and analysis of network parameters. The network trained on all seven MC patients was then tested on five AUH patients in order to check its generalisability across institutions. All patients were diagnosed with localization-related epilepsy and had typical focal impaired awareness seizures (FIAS) with focal to bilateral tonic-clonic seizures (FBTCS) in two patients (ILAE 2017 seizure classification^21^). All MC patients underwent surgery with a good surgical outcome after the two-year follow-up period (Engel’s Class 1). Information on surgery and surgical outcome was not available for the AUH dataset.

Subdural grid and/or intracerebral electrode placement was dictated strictly by clinical requirements and therefore electrode configuration varied between patients. Intracranial EEGs from multiple sites of temporal, frontal and parietal cortices as well as the amygdala-hippocampus complex were recorded at the sampling rate of 500 Hz with the number of channels varying from 16–108 across patients. Demographic and clinical characteristics of patients as well as the amount of data used in our study are presented in Table 1.

**Table 1:** Patients’ demographic and medical data.

Patient	Gender	Age at admission	Age at onset of seizures	Seizure Type	iEEG length spanned by interictal intervals analysed	Total No. of seizures in the spanned iEEG	Total length of interictal intervals analysed	Total No. of channels (No. of bad channels)
MC1	M	26	21	Focal Complex/Partial (FIAS)	86 h	150	8 h	16 (1)
MC2	M	33	23	Focal Complex/Partial (FIAS)	45 h	6	7 h	60
MC3	M	37	23	Focal Complex/Partial (FIAS)	90 h	26	6 h	84 (2)
MC4	M	16	13	Focal Partial-Secondary generalised tonic-clonic (FBTCS)	103 h	9	8 h	108
MC5	M	16	1	Focal Complex/Partial (FIAS)	21 h	3	6 h	88
MC6	M	9	9	Focal Complex/Partial (FIAS)	55 h	21	8 h	96 (7)
MC7	M	58	55	Focal Partial-Secondary generalised tonic-clonic (FBTCS)	30 h	2	9 h	88 (2)
AUH1	M	31	30	Focal Complex/Partial (FIAS)	71 min	2	10 min	94
AUH2	M	52	50	Focal Complex/Partial (FIAS)	18 min	4	10 min	70
AUH3	F	21	16	Focal Complex/Partial (FIAS)	27 min	2	10 min	69
AUH4	F	55	50	Focal Complex/Partial (FIAS)	12 min	2	10 min	46
AUH5	F	15	12	Focal Complex/Partial (FIAS)	38 min	4	10 min	68

Seizure types as per the ILAE 2017 Operational Classification are given in parentheses. FIAS – focal impaired awareness seizure; FBTCS – focal to bilateral tonic-clonic seizure.

### Selection of electrographic events as the ‘ground truth’

To obtain a more complete representation of interictal activity in each patient, we used the following criteria to select segments for analysis. First, we scanned the whole record available in each patient and selected segments free of gross artifacts such as large deviations from the zero line (possibly due to patient’s movements or technical problems). Then for each MC patient we selected 6–9 one-hour segments of interictal activity (regardless of the sleep-wake cycle). Because records from the AUH dataset were significantly shorter, we were able to extract 10-min segments of interictal activity from each patient. For both datasets, the only criterion was that interictal segments had to be separated by at least one hour from the preceding and the following seizures. Also, in order to incorporate possible modulations in the interictal patterns as much as possible in the MC dataset, we aimed to select segments from different parts of the whole record which could span up to several days. As a result, the selected segments could be separated from each other by several hours and together covered many hours of recordings (from 21 to 103 hours from the beginning of the first segment to the end of the last segment depending on the total length of the record; Table 1).

Signal pre-processing for both datasets included a check for and removal of any missing data points and highpass filtering at 0.5 Hz (300-order FIR, forward-backward filtering). If a distinctive line interference was present in the signal, notch filtering was also applied (a second order IIR notch filer, Q factor = 35). A continuous power spectrogram (short-time FFT with the Hanning window) of the pre-processed data was calculated with time and frequency resolution of 0.25 s and 4 Hz, respectively. The spectrogram was then averaged over frequencies within the following eight spectral bands: theta (4–8 Hz), alpha (8–13 Hz), beta (13–30 Hz), gamma1 (30–56 Hz), gamma2 (64–116 Hz), rip1 (124–176 Hz), rip2 (184–196 Hz) and rip3 (204–236 Hz) giving the eight spectral power values used as features in further analysis. The division of the ripple band (>124 Hz) into three sub-bands (rip1, rip2 and rip3) was necessary in order to avoid frequencies of 120, 180 and 200 Hz at which interference noise was sometimes present.

To find events which would be considered as a ground truth for network training and testing, we used an automated threshold-based approach to find event candidates. Those candidates were then visually verified for their inclusion into or exclusion from the ground truth set of events. The initial screening for spike, ripple and RonS candidates was done using the following method. For each spectral band in one-hour spectrogram, we calculated the empirical probability density distribution of the log-transformed power values. The logarithm of power density is known to be well approximated by normal distribution^22^. In our case this was also confirmed by the Kolmogorov-Smirnov normality test (p < 0.05). The maximum (peak) value of this normal distribution is the *mode* i.e., the most likely (or most probable) value of spectral power. However, due to the discrete nature of binning when the empirical distribution is calculated, the position of its peak may be slightly displaced from the exact mode due to random variations from bin to bin. To reduce this effect, we calculated the *mean* of the most probable log-transformed power values that constituted 20% of all distribution. In other words, we took a mean value in the 20% vicinity of the peak of the power density distribution. This value was taken as representing the *baseline* value of spectral power when the events of interests (spikes and ripples) are not present. All power values were then normalized by dividing them by the corresponding baseline power for each spectral band. These *relative* spectrograms were used for visual reviews of the event candidates.

To find event candidates within each time bin (0.25 s), we used the following threshold values applied to the relative spectrograms. To be identified as a spike, the spectral power had to be at least 4 times the baseline power in *both* beta and gamma bands but not in the ripple band (the ‘spike’ criterion: a 4-fold increase in power in the beta-gamma bands relative to the baseline power). For ripples, the spectral power had to be at least 7 times the baseline power within at least one ripple sub-band (rip1 or rip2 or rip3) but not in the beta-gamma bands (the ‘ripple’ criterion). For RonS, both the ‘spike’ and the ‘ripple’ criteria had to be satisfied. Lastly, the baseline candidates were selected as time bins where none of the above criteria was met. The higher threshold for ripples compared to spikes (7 versus 4) was chosen based on our preliminary analysis showing that the relative increase in power during ripples was indeed higher than the power changes during spikes.

All final ground truth events were selected from the candidates using visual inspection and verification at the appropriate temporal resolution (for spikes) and with the help of additional highpass filtering of iEEG at >100 Hz for ripples and RonS. HP filtering allowed us to make sure that the number of oscillations within a HFO burst is more than 3 to be qualified as a ripple. This criterion helps avoid ‘false’ HFOs which are a by-product of highpass filtering of spikes^23,24^. After visual inspection of approximately 1200–1300 event candidates of each class (spike, ripple, RonS and baseline), we selected 1000 events (per class) for every patient from the MC dataset. Importantly, these 1000 events (per event class and per patient) were randomly selected from all channels and all one-hour segments in each MC patient.

### The LSTM network

**Figure 1:**
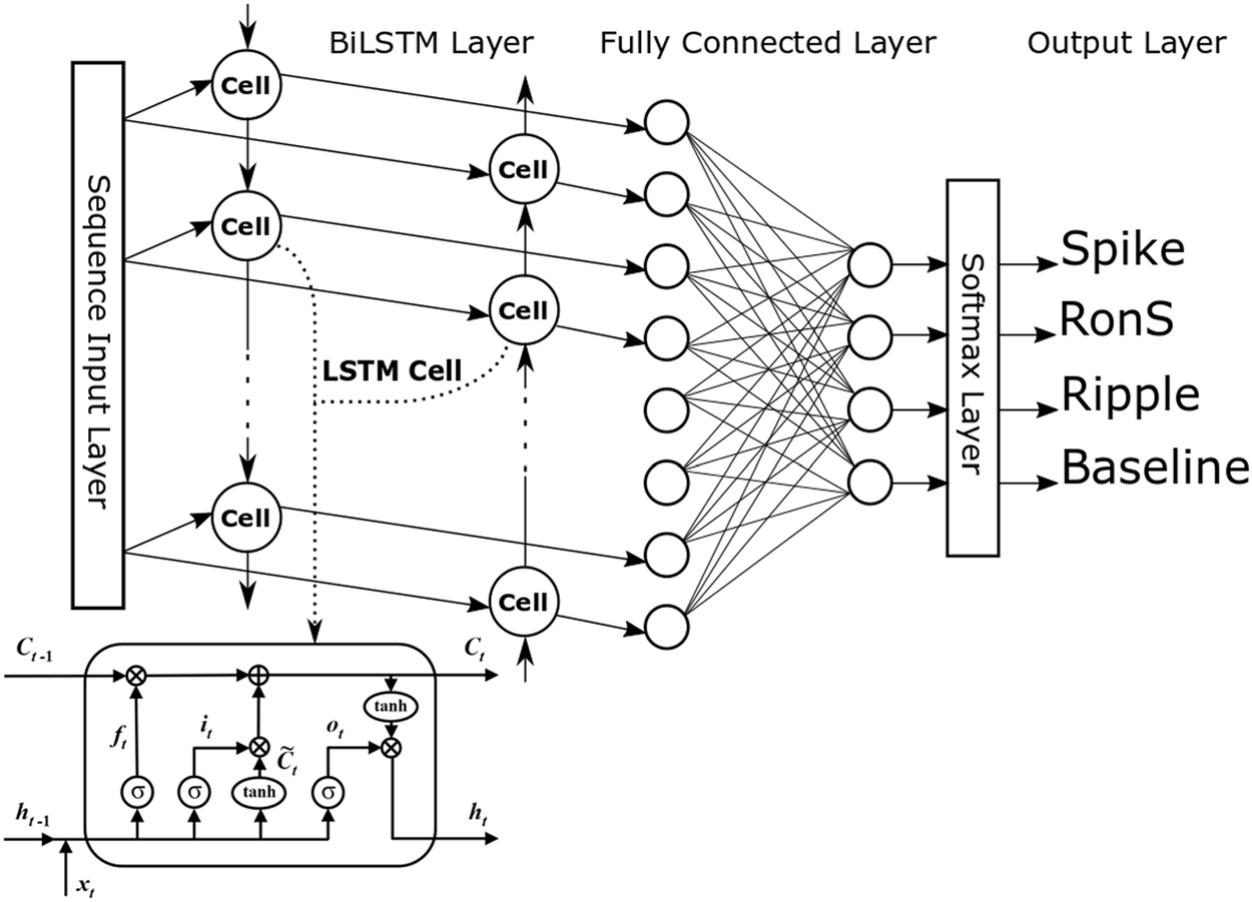
The LSTN network architecture based on one bidirectional LSTM layer.

The major component of all LSTM networks is a hidden layer (the LSTM layer) consisting of the memory cells. Each memory cell has an internal structure including the cell itself and three gates which control the cell behaviour across time. The cell gates are essential for the ability of the cell to act as a memory unit, and they allow the network to detect long-term dependencies in the stream of input data. The input comes to the network through a sequence input layer and after the LSTM layer there are a fully connected layer and the output layer (usually realizing the softmax operation) which conclude the classification architecture of the network. The network for this study was created using the MATLAB version 2018b (MathWorks, Inc., Natick, MA). The main architecture was based on one bidirectional LSTM layer (Fig. 1). The bidirectional layer of memory cells allows the network to analyse information in both directions (from past to future and backwards) and this allows the network to understand the context better. As part of our exploration of the network parameter space, we also used several modifications of the network architecture namely, a single LSTM layer or two consecutively connected LSTM layers as well as dropout layers after each LSTM layer.

As an input to the network, we used relative spectral power values within all 8 frequency bands for each time bin as well as the preceding and the following bin. Thus, the feature input vector had a dimension of 8 × 3 (the number of frequency bands by the number of time points). Three consecutive time bins served to avoid a possible ‘split’ of the event between two adjacent bins as well as to provide the temporal pattern and context of the events to the network. Network training was performed on the MC dataset using different scenarios as follows: (1) training and testing within each MC patient (within-subject validation); (2) training for one patient’s data and testing on other patients (between-subjects validation); (3) training and testing on all MC patients (‘global’ training and testing); and finally (4) the ‘global’ network trained on the whole MC dataset was tested on each patient from the AUH dataset (‘between-institutions’ validation). All training sessions were performed 10 times using random selection of 50–500 events (per class) from the ground truth set while other 500 events (per class) were used for testing. Network performance was assessed by the mean values and standard deviations (in %) for total accuracy, *Acc* across all four classes of events as well as sensitivity, *Sens(i)* and specificity, *Spec(i)* for each class *i* = 1 ÷ 4. These values were calculated using the following formulae:

$${\rm{Accuracy}}:\,Acc=100\times \frac{{\sum }_{i=1}^{4}\,TP(i)}{{\sum }_{i=1}^{4}\,P(i)}$$

$${\rm{Sensitivity}}\,{\rm{or}}\,{\rm{true}}\,{\rm{positive}}\,{\rm{rate}}:Sens(i)=100\times \frac{TP(i)}{P(i)}$$

$${\rm{Specificity}}\,{\rm{or}}\,{\rm{true}}\,{\rm{negative}}\,{\rm{rate}}:\,Spec(i)=100\times (1-FPR(i))=100\times (1-\frac{FP(i)}{N(i)})$$

here, *P(i)* and *N(i)* are the numbers of real positive and negative cases in the data for each class, *TP(i)* and *FP(i)* are the numbers of true positives (hits) and false positives (false alarms) for each class, *FPR(i)* is the false positive rate.

## Results

### Waveforms of the ground truth events

**Figure 2:**
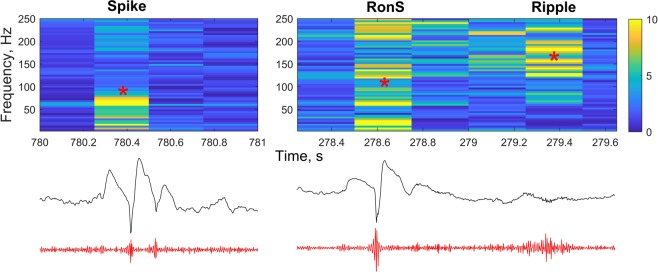
Representative spike (left) as well as RonS and ripple (right) detected by our threshold method and confirmed by visual analysis. Top, relative spectrograms; middle, raw iEEG; bottom, highpass filtered iEEG at 100 Hz. Note the lack of spectral power in the ripple bands and a lower amplitude of fast oscillations in the filtered iEEG for the spike. These oscillations are due to the effect of highpass filtering of spike and represent a ‘false’ ripple.

Representative spike, RonS and ripple detected by our threshold method and confirmed by two experts (A.V.M. and A.M.M.) using visual and spectral analysis are shown in Fig. 2. The problem with spikes is that highpass filtering of a spike produces a burst of high frequency oscillations which can be mistakenly marked as ripple resulting in a false positive error for ripples and RonS events. This is a well-known effect of highpass filtering of sharp waves producing sharp transients^23^. The spectrogram helps avoid this problem because sharp transients of a spike or sharp wave do not usually have a significant spectral power in the ripple bands (>120 Hz), and using a threshold of 7 helped us tell apart single spikes (Fig. 2, *left*) from RonS (Fig. 2, *right*).

The major focus of this study was to explore the ability of the network to generalise (a) across different segments of iEEG within each patient (within-subject validation); (b) across different patients from the same dataset (between-subjects validation) and finally, (c) whether the network with optimal parameters can generalise across datasets from different institutions (‘between-institutions’ validation). In the majority of calculations we used a bidirectional LSTM network with the number of hidden units (HU) equal to 200. Non-overlapping training and testing sets each contained 500 events per class (spike, ripple, RonS and baseline) randomly selected from the ground truth set with the number of randomizations equal 10.

### Within-subject and between-subjects validations

During the within-subject validation, network testing showed high values of accuracy exceeding 90% for spikes, ripples and baseline segments for all patients (the main diagonal entries in Table 2). The results of RonS detection were slightly lower in accuracy being in the range 85–90% for 4 out of 7 patients in the MC dataset (Table 2).

**Table 2:** Network accuracy (%) for within-subject validation (the main diagonal entries shown in *italics*) and between-subjects validation (the off-diagonal entries, patient’s data used for training/testing are along the columns/rows, respectively).

Spikes	MC1	MC2	MC3	MC4	MC5	MC6	MC7
MC1	*95.6 (1.4)*	88.3 (5.5)	95.6 (1.4)	**84**.**5 (2**.**1)**	93.1 (2.7)	**81**.**1 (3**.**8)**	93.2 (2.8)
MC2	99.4 (0.3)	*98.2 (2.0)*	99.7 (0.1)	98.1 (1.3)	97.9 (2.4)	99.0 (0.8)	99.7 (0.1)
MC3	97.3 (0.2)	86.0 (2.5)	*92.8 (8.2)*	93.4 (0.6)	92.5 (0.6)	**79**.**3 (2**.**7)**	**82**.**9 (3**.**2)**
MC4	97.1 (0.8)	88.1 (4.4)	98.6 (0.1)	*94.7 (3.2)*	96.2 (0.3)	87.9 (1.0)	93.5 (0.7)
MC5	98.6 (0.5)	93.2 (1.1)	99.0 (0.3)	94.9 (0.7)	*96.6 (2.8)*	92.3 (1.0)	97.7 (0.7)
MC6	99.4 (0.3)	98.7 (0.4)	99.8 (0.1)	99.3 (0.4)	99.1 (0.4)	*97.4 (1.1)*	99.2 (0.3)
MC7	98.3 (0.1)	95.0 (1.1)	99.5 (0.4)	96.8 (2.0)	95.6 (0.9)	92.3 (2.1)	*93.5 (1.6)*
**RonS**							
MC1	*89.1 (5.5)*	97.6 (1.1)	95.5 (4.4)	97.1 (2.4)	93.1 (3.3)	98.7 (0.7)	93.1 (1.6)
MC2	**75**.**1 (0**.**5)**	*94.0 (4.2)*	**46**.**2 (8**.**2)**	**57**.**3 (16**.**6)**	**75**.**8 (3**.**5)**	88.4 (2.0)	**55**.**7 (5**.**2)**
MC3	**78**.**4 (8**.**1)**	**61**.**5 (2**.**4)**	***85****.****4 (2****.****8)***	**84**.**5 (1**.**8)**	**72**.**1 (10**.**2)**	89.3 (1.3)	95.6 (0.3)
MC4	92.4 (1.5)	92.9 (1.6)	89.1 (4.1)	*87.9 (3.8)*	**78**.**3 (6**.**5)**	97.2 (0.1)	**81**.**2 (12**.**2)**
MC5	88.7 (3.2)	90.8 (7.4)	**83**.**0 (2**.**6)**	**83**.**6 (3**.**7)**	*94.8 (4.5)*	94.0 (4.5)	**83**.**0 (4**.**0)**
MC6	**77**.**6 (9**.**4)**	91.3 (7.2)	**58**.**7 (0**.**4)**	**65**.**0 (7**.**6)**	**70**.**2 (18**.**7)**	*90.8 (9.3)*	**53**.**6 (4**.**8)**
MC7	**68**.**2 (3**.**6)**	**68**.**0 (7**.**9)**	**54**.**4 (2**.**6)**	**55**.**6 (1**.**1)**	**74**.**5 (0**.**9)**	**84**.**2 (4**.**8)**	***85****.****0 (10****.****2)***
**Ripples**							
MC1	*96.7 (1.0)*	93.9 (0.1)	94.4 (1.7)	93.0 (2.0)	96.9 (2.1)	90.9 (6.1)	**73**.**0 (1**.**7)**
MC2	98.6 (0.5)	*95.1 (0.7)*	**77**.**3 (7**.**5)**	93.7 (3.5)	90.7 (6.4)	93.0 (6.8)	**77**.**2 (4**.**0)**
MC3	98.8 (0.5)	99.1 (0.4)	*95.2 (4.8)*	95.7 (0.7)	97.6 (0.7)	96.8 (1.4)	**83**.**1 (3**.**5)**
MC4	98.2 (0.5)	96.5 (2.1)	97.2 (0.3)	*96.3 (2.7)*	98.6 (0.3)	93.9 (5.2)	87.3 (6.4)
MC5	97.5 (0.3)	94.0 (2.0)	96.4 (0.6)	96.2 (1.4)	*95.0 (0.6)*	94.6 (4.5)	**74**.**4 (0**.**3)**
MC6	98.7 (1.5)	96.8 (2.3)	90.7 (12.0)	95.7 (3.5)	96.2 (4.7)	*93.3 (8.1)*	**83**.**9 (11**.**2)**
MC7	99.5 (0.2)	99.4 (0.1)	**82**.**9 (16**.**0)**	96.1 (3.5)	96.1 (2.2)	93.3 (8.3)	*93.2 (7.4)*
**Baseline**							
MC1	*98.3 (0.4)*	95.3 (0.7)	99.6 (0.3)	99.1 (0.7)	99.6 (0.1)	98.1 (0.1)	98.4 (0.3)
MC2	99.8 (0.3)	*97.5 (1.3)*	100.0(0.1)	99.5 (0.1)	99.9 (0.2)	99.3 (0.1)	99.0 (0.3)
MC3	98.7 (0.3)	93.4 (0.9)	*99.1 (1.0)*	98.8 (0.6)	99.5 (0.2)	97.1 (0.1)	98.5 (0.1)
MC4	99.2 (0.3)	95.3 (0.7)	99.7 (0.1)	*99.3 (0.7)*	99.8 (0.1)	98.2 (0.1)	98.7 (0.4)
MC5	98.0 (0.2)	93.5 (1.3)	99.4 (0.1)	98.4 (0.6)	*99.2 (0.9)*	96.5 (1.0)	97.8 (0.6)
MC6	99.1 (0.1)	95.8 (0.3)	99.8 (0.1)	99.0 (0.3)	99.8 (0.1)	*98.2 (1.4)*	98.5 (0.7)
MC7	99.1 (0.2)	96.2 (1.4)	99.9 (0.1)	99.3 (0.4)	99.7 (0.2)	98.8 (0.9)	*99.3 (0.1)*

Means and standard deviations are calculated from 10 randomizations with 500/500 events for training/testing. Values of accuracy below 86% are shown in bold. Network parameters: No. of hidden units (HU) = 200; 1000 iterations for training.

During the ‘between-subjects’ validation, the network was trained on each patient’s data and then tested on all other patients within the MC dataset. Here the network showed poorer results with accuracy below 86% in 4 and 7 cases for spikes and ripples, respectively (out of 42 pair-wise comparisons per class) as well as below 80% in 19 and below 60% in 7 out of 42 pair-wise comparisons for RonS (the off-diagonal entries in Table 2). This was the evidence that a network trained on one patient does not necessarily generalise when applied to other patients, and this is likely due to inter-individual variability of electrographic waveforms especially for RonS events (Table 2).

### Training and testing of a ‘global’ network

To overcome a limited ability of the network to generalise from one patient to another, we decided to train a network on all patients from the MC dataset. Thus, the global feature vector 1000 × 4 × 7 = 28000 was created (1000 events per event class per patient). Then the network training and testing on this global vector was done in a similar way by random selections of 500 events (per class) from each MC patient. These 500 × 4 × 7 = 14000 events were used for training while the other 14000 events were used for testing, and the process was repeated 10 times. For the ‘between-institutions’ validation, the ‘global’ network was tested on all 10-min interictal segments from the AUH dataset. The ‘global’ network was able to recognize all events from both datasets with the total accuracy and specificity greater than 90% in all cases and sensitivity less than 86% in only two cases (for spikes in patient MC6 and ripples in patient MC7, Table 3). Overall, the average sensitivity for spikes and RonS was slightly lower (91.3 ± 5.1% and 92.5 ± 4.1%, respectively) compared to ripples (96.7 ± 4.9%).

**Table 3:** Results of the ‘global’ network testing for all patients from two institutions.

Patient ID	Total Accuracy	Spikes		RonS		Ripples		Baseline	
		Sensitivity	Specificity	Sensitivity	Specificity	Sensitivity	Specificity	Sensitivity	Specificity
MC1	95.3 (1.9)	96.5 (0.1)	98.3 (0.2)	87.6 (7.4)	98.0 (0.2)	97.5 (0.7)	97.3 (2.5)	99.5 (0.4)	100 (0.0)
MC2	92.4 (1.1)	88.1 (3.0)	99.9 (0.1)	89.7 (2.4)	95.8 (1.1)	98.2 (0.1)	94.1 (0.3)	93.4 (1.1)	99.9 (0.1)
MC3	95.4 (1.7)	95.0 (4.0)	98.5 (0.1)	90.3 (0.7)	97.5 (2.2)	96.8 (3.4)	98.4 (0.9)	99.6 (0.1)	99.5 (0.8)
MC4	95.3 (0.9)	92.6 (0.9)	99.1 (0.3)	94.0 (0.6)	96.0 (1.5)	95.1 (5.0)	98.7 (0.5)	99.6 (0.1)	99.9 (0.1)
MC5	95.2 (1.5)	93.3 (5.8)	99.7 (0.3)	89.0 (0.3)	97.8 (1.4)	99.2 (0.6)	96.1 (0.9)	99.1 (0.4)	99.9 (0.1)
MC6	93.3 (0.1)	**82**.**5** (**1**.**3)**	99.9 (0.1)	96.8 (0.9)	93.5 (0.7)	96.3 (1.8)	97.7 (0.9)	97.6 (0.1)	99.9 (0.1)
MC7	91.3 (3.7)	87.9 (2.4)	99.3 (0.2)	96.9 (3.0)	90.1 (6.0)	**81**.**9** (**9**.**7)**	99.0 (0.9)	98.6 (0.3)	99.9 (0.1)
AUH1	97.5 (0.1)	90.0 (0.9)	99.8 (0.1)	94.4 (0.8)	99.9 (0.1)	98.2 (0.2)	97.7 (0.1)	97.5 (0.1)	99.9 (0.1)
AUH2	96.5 (0.1)	86.1 (3.7)	99.9 (0.1)	89.7 (3.8)	99.9 (0.1)	99.5 (0.1)	96.5 (0.1)	96.4 (0.1)	99.9 (0.1)
AUH3	98.7 (0.1)	96.2 (1.0)	99.9 (0.1)	89.4 (1.8)	99.9 (0.1)	99.5 (0.3)	98.8 (0.1)	98.7 (0.1)	100 (0.0)
AUH4	99.7 (0.1)	99.8 (0.2)	99.8 (0.1)	100 (0.0)	100 (0.0)	100 (0.0)	99.8 (0.1)	99.7 (0.1)	100 (0.0)
AUH5	97.5 (0.1)	87.5 (5.9)	99.9 (0.1)	N/A	N/A	97.9 (0.2)	97.5 (0.1)	97.5 (0.1)	99.9 (0.1)

The mean values (%) and standard deviations (in parentheses) are presented. Values below 86% are shown in bold. Note: RonS events in patient AUH5 were not found (marked by N/A).

### Network behaviour in the parameter space

**Figure 3:**
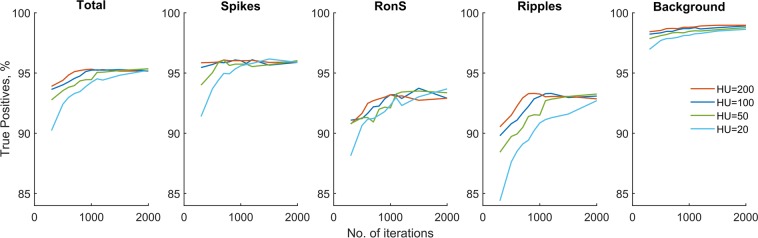
The LSTM network performance as a function of the number of hidden units (HU) and the number of iterations during training (the same training and testing sets were used at different parameters of the network).

Finally, we explored the network behaviour as a function of the most important network parameters such as the number of hidden units (bidirectional LSTM; HU = 20 ÷ 200), the size of the training set (N = 50 ÷ 500 events per class per patient) and the number of iterations during training (NI = 200 ÷ 2000). For all these parameters which determine the network size and its trained state, testing was always performed on 500 events (per class per patient) not used in training. At N = 500, the network accuracy and sensitivity reached a maximum level at about 1000 iterations and at this level it practically plateaued for the next 1000 iterations for all event classes (Fig. 3). A slight decrease in sensitivity (most likely due to overfitting) was observed only for RonS and ripples from 1000 to 2000 iterations (Fig. 3). This result demonstrates the network robustness to overfitting.

In regard to the number of hidden units, the network showed a slight decline in sensitivity from HU = 200 to HU = 50 with a more noticeable decline at HU = 20. Remarkably, with only 20 hidden units the network was able to detect more than 90% of events correctly with the number of iterations greater than 1000 (Fig. 3).

A decrease in size of the training set from N = 500 to N = 50 also had only a minimal effect on the network performance with a drop in sensitivity for RonS from 92.04% to 86.89% (statistically significant by paired t-test, p = 0.03; Table 4). Changing the network structure (one LSTM layer versus two consecutive layers with or without dropout layers) did not have any noticeable effect on performance compared to the bidirectional LSTM network (a standard model in this study; Table 4).

**Table 4:** Network performance at different sizes of the training set (A) and with a different structure of its hidden layer(s) (B).

	Sensitivity (True Positive Rate, TPR) by event class (%)				Accuracy (%)
	Spikes	RonS	Ripples	Baseline	Total
**A**. **Training** **set (N)**					
50	91.9 (2.7)	84.1 (2.8)*	92.2 (5.8)	98.0 (0.1)	91.6 (1.4)
100	91.6 (0.5)	88.4 (4.0)*	93.7 (4.0)	97.6 (1.0)	92.8 (1.9)
250	91.6 (1.0)	91.3 (0.2)	94.9 (4.3)	98.1 (0.3)	94.0 (1.2)
**500**	**90**.**8 (0**.**2)**	**92**.**0 (0**.**6)**	**95**.**0 (3**.**9)**	**98**.**2 (0**.**3)**	**94**.**0 (1**.**1)**
**B**. **Network** **structure**					
One LSTM	96.1 (0.4)	92.5 (0.5)	95.5 (3.6)	98.6 (0.1)	95.7 (0.8)
Two LSTMs	95.8 (0.1)	92.6 (1.0)	95.8 (3.5)	99.0 (0.2)	95.8 (0.6)
Two LSTMs + Dropouts (0.2)	96.3 (0.7)	92.4 (0.9)	96.0 (3.4)	98.9 (0.2)	95.9 (0.7)
BiLSTM	96.3 (0.3)	91.9 (1.8)	95.8 (3.6)	98.7 (0.1)	95.7 (0.5)

For both A and B: HU = 200; NI = 1000. *Asterisks mark a significant decrease in performance with smaller training sets (N = 50 and N = 100) compared to N = 500 (paired t-test, p = 0.03). N = 500 was used as a standard training set (shown in bold).

## Discussion

In this study we applied for the first time Long Short-Term Memory deep learning networks for detection of electrographic events such as epileptic spikes, ripples and ripples-on-spikes in intracranial EEG records from patients with intractable epilepsy. The LSTM network was trained on the ground truth set of events which was initially created through a novel automated threshold-based approach with subsequent visual verification and selection of events of four classes namely, spikes, ripples and ripple-on-spike as well as baseline activity.

Our threshold method for automated detection of events is different from the commonly used methods in that it avoids calculation of the standard deviation of spectral power as well as it operates on the relative values of spectral power (relative to baseline). The standard deviation is used in the traditional methods to calculate threshold as the mean power (or RMS) plus several (e.g., 4 or more) standard deviations. However, if the standard deviation is calculated over the entire EEG segment, it would be unavoidably inflated due to the presence of the events of interest (spikes and ripples). These events are rare but have a higher amplitude than baseline activity and therefore may significantly skew the (log-transformed) power density distribution toward higher values. This would result in an inflated standard deviation and an unnecessary enhancement of the threshold. As a result, many events of interest may be missed. This problem can be addressed if the standard deviation is calculated not over the entire segment but over the periods of baseline activity (i.e., when the events of interest are not present). But this requires finding those events in the first place. Our method also avoids manual or automated selection of baseline segments which would ideally lack any events of interest. Instead, the mean spectral power of baseline activity is calculated from the empirical distribution of spectral power density by taking the mean of the *most probable* spectral power values. This is justified under the assumption that relatively rare events of interest, although skewing the power density distribution at the high end, do not significantly affect the most probable values around the peak of this distribution. Indeed, the most probable power values are determined by baseline activity which constitutes up to 80–90% of activity (by duration) during interictal or even preictal periods.

After the calculation of the baseline power within each spectral band, our method uses relative thresholds for a *ratio* between the current-bin spectral power and the corresponding baseline power (for each spectral band). Specific values of these relative thresholds used in this study were selected on the basis of our preliminary analyses comparing our threshold method with the visual inspection of the events.

We are aware of only a few studies where deep neural networks were applied to the study of epileptiform events such as spikes or HFOs. The convolutional neural network (CNN) was used by Johansen *et al*. (2016) for detecting epileptic spikes in scalp EEG from five patients^25^. The authors report that the CNN achieved the area under the ROC curve (AUC) of 0.947. This was higher than the best performing model based on a Support Vector Machine which achieved an AUC of 0.912^25^. The CNN was also used by Zuo *et al*. (2019) to detect ripples and fast ripples in iEEG recordings from 6 patients with intractable epilepsy. The authors report that the CNN was able to detect HFOs with a higher sensitivity (77.04% for ripples and 83.23% for fast ripples) and specificity (72.27% for ripples and 79.36% for fast ripples) than four traditional automated methods implemented in the RIPPLELAB toolbox^26^. Guo *et al*. (2018) employed the stacked sparse auto encoder (SSAE) to facilitate the clinical detection of HFOs in the MEG records from 20 patients with localization related epilepsy. The proposed SSAE detector also outperformed the threshold-based models by achieving 89.9% in accuracy, 88.2% in sensitivity and 91.6% in specificity^27^. The LSTM architecture is better suited for the analysis of time series data compared to other neural network architectures and the LSTM networks have been started to attract interest in the field of EEG analysis. Thus, a LSTM recurrent network has been recently used for recognition of human emotional states^28^ and for human decision prediction^29^ from scalp EEG with the reported results outperforming the traditional analytic machine learning algorithms.

In this study, we applied for the first time a LSTM network for the detection of distinctive electrographic events such as epileptic spikes, ripples and ripples-on-spikes. After creating a ground truth set of events through our threshold method and subsequent confirmation of events by visual analysis, the LSTM network was trained and tested on different subsets of data from individual patients. The main results can be summarized as follows. First, the LSTM network trained on the data from an individual patient can detect other events within the same patient with high accuracy but would show a poorer performance on other patients. It is most likely due to individual variations in spectral and temporal profiles of the events across patients. A limited generalisability is a phenomenon when a network trained within one dataset shows a worse performance on other datasets. It is one of the problems that can limit the application of artificial neural networks in many fields of science and technology. The lack of generalisability is often a result of overfitting when a network is over-trained on a specific set of data features which makes this network well-tuned and over-sensitive to those features only. One powerful solution to avoid overfitting is the inclusion of dropout layers into the network architecture. The dropout layer forbids weight adjustment on some randomly selected units within the hidden layer on each training step thus preventing overfitting. In this study we did not see any prominent effects of overfitting within the explored range of network parameters (Fig. 3) and thus we attribute a poor ‘between-subjects’ generalisability of the network trained just on one patient to the individual variation of the electrographic events (such as the amplitude and duration of spikes and ripples which can vary from patient to patient). The important step to achieve a good generalisability in our study was to train the network on all seven patients from the MC dataset. Also of importance, the ground truth training set was created by events randomly selected from all channels in each MC patient thus increasing the intra- and inter-subject variability of data features in the training set. The network trained on seven MC patients was able to recognize other events in the same patients as well as in the dataset from a different institution (the AUH dataset) with high accuracy, sensitivity and specificity (Table 3). A slightly lower sensitivity for spikes and RonS compared to ripples across two datasets is likely due to a greater variability of spike amplitude which varies considerably within and between patients. This result emphasises the importance of creating a *fully representative* training set for supervised learning of neural networks which would cover a broad range of feature variations across many individuals and different institutions.

Our results demonstrate that the LSTM deep learning networks can be used for automated detection of epileptiform events such as spikes, RonS and ripples within intracranial EEG records. Most importantly, the network trained on an extended dataset from 7 patients showed excellent generalisability detecting events with high accuracy, sensitivity and specificity in all records from two independent datasets. Deep learning networks can significantly accelerate the analysis of prolonged iEEG data and increase its diagnostic value as well as provide additional information for a more accurate localization of the seizure onset zone with potential improvement of surgical outcomes.

## Data Availability

The data of the secondary analysis in this study are available on request from the corresponding author (A.V.M.). The original iEEG data are publicly available on the IEEG portal (www.ieeg.org).
